# Payors, Caregivers, and Teens: Aligning Priorities for Effective Digital Mental Health Tools

**DOI:** 10.2196/72587

**Published:** 2025-08-14

**Authors:** Jennifer Huberty, Jacqlyn Yourell, Lara Baez, Louisa Salhi

**Affiliations:** 1 Fit Minded Inc., 2901 E Greenway Road, PO Box 30271, Phoenix, AZ, 85046, United States, 1 6029356986; 2 Kooth Digital Health, London, United Kingdom; 3 School of Psychology, Keynes College, University of Kent, Canterbury, United Kingdom

**Keywords:** payors, parents, adolescents, mental health, digital mental health interventions, caregivers, teens

## Abstract

Digital mental health (DMH) tools are a promising solution to the growing need for mental health care among teens because of their scalability and accessibility. Yet the key stakeholders—payors (who reimburse), caregivers (who facilitate adoption and access), and teens aged 13‐17 (the primary users of these tools)—often have conflicting priorities. These misaligned priorities can limit investment in tools that align with user needs and promote long-term engagement, ultimately hindering the effective deployment of DMH tools for teens. This viewpoint paper outlines payor, caregiver, and teen priorities for DMH tools and proposes novel strategies to align these priorities. We argue that this alignment is critical for driving meaningful improvements in teen well-being.

## Introduction

The use of digital mental health (DMH) platforms has grown significantly, with increasing numbers of teens using these tools for support. Specifically, health-related app usage among those aged 14 to 22 was nearly 70% in 2020, showing an increase from 2018, particularly in apps focused on mental health issues such as stress and depression [1]. Digital tools such as apps, teletherapy, and artificial intelligence–driven platforms are transforming the way teens access mental health care [2]. These tools offer scalable and accessible ways to address a variety of mental health needs [3], making them an attractive solution for a range of stakeholders: for payors, they offer cost-efficiency; for caregivers, increased access and support for their child; and for teens, convenient, on-demand help.

A payor is an organization (eg, an insurance company) that covers health care costs, including mental health care such as therapy and counseling. In this paper, we use the term “caregivers” broadly to include primary caregivers and legal guardians who play a central role in supporting teens’ adoption and use of DMH tools. Teens in this paper are individuals aged 13 to 17, similar to the age range covered under pediatric mental health benefits by both public and private US insurance programs [4-6].

Payor, caregiver, and teen priorities in DMH are not aligned. Payors prioritize cost-effectiveness, scalability, and regulatory compliance, relying on standardized clinical outcomes to demonstrate return on investment (ROI) [7]. Caregivers and teens value safety, privacy, expert validation, and meaningful engagement, with teens particularly emphasizing autonomy, connectedness, and immediate support over traditional clinical measures [3-5]. Understanding and addressing competing priorities is essential to designing and implementing scalable DMH tools for teens and their families that can be integrated into a complex mental health care setting.

In this paper, we argue that greater attention must be paid to what users—teens and caregivers—actually want and need. By highlighting the perspectives and priorities of teens and caregivers, we suggest strategies to align these with payors’ objectives, encouraging payors to consider these needs when making decisions about which DMH solutions to cover and reimburse.

## Payors’ Priorities in Digital Mental Health

Payors are crucial stakeholders because they determine which mental health programs (eg, therapy practices, and DMH tools) are accessible to their policyholders based on financial viability. In the absence of reimbursement from payors, providers (eg, clinicians, clinics, or organizations that deliver mental health services) struggle to offer programs because users cannot afford them. Payors prioritize scalability and regulatory compliance, with interventions that meet privacy and security standards (eg, Health Insurance Portability and Accountability Act–HIPAA) more likely to gain approval and be integrated into broader health care plans [8]. Payors’ primary concern in DMH is ensuring that interventions are both cost-effective (delivering optimal outcomes relative to the cost) and yield measurable outcomes that demonstrate ROI, in which the financial benefits, such as reduced long-term costs, outweigh the initial investment [7]. With the rising cost of health care, payors are increasingly prioritizing solutions that improve patient outcomes and also reduce the overall cost of care such as preventive measures and early interventions that help avoid expensive treatments such as emergency room visits and hospitalizations [9].

In order to demonstrate cost-effectiveness and ROI, payors rigidly prioritize data-driven evidence tied to clinical symptoms such as depression and anxiety, neglecting to consider critical mental health outcomes that matter the most to users, such as stress, sleep, daily functioning, burnout, and quality of life [10]. Improving these broader outcomes has system-wide benefits, such as increased engagement in education and future employment, which further contribute to long-term economic gains [11]. Compounding this issue is the reliance on widely used standard measures for depression and anxiety (eg, Patient Health Questionnaire [PHQ-9] and General Anxiety Disorder [GAD-7]) and flawed cutoff scores that fail to capture the complexities and nuances of mental health challenges faced by teens in today’s society [12,13,14]. Furthermore, payors exclude teens from coverage who do not meet full diagnostic criteria or those who experience subthreshold symptoms and could benefit from DMH treatment [15,16]. Payors’ narrow view of what constitutes a worthwhile DMH tool restricts investment in innovative solutions that may better align with the preferences of teens and their caregivers.

Notably, sustained engagement with and adherence to DMH tools is low for teens [3]. This is influenced by several factors—for example, teens often use these tools during a mental health challenge or crisis [17]; yet, the tools typically target clinical outcomes (eg, depression and anxiety) that may not align with their immediate concerns. To improve engagement, digital tools need to target the outcomes that teens and caregivers care about most, rather than focusing solely on traditional symptom measures [18]. To truly advance mental health care, payors must expand their scope to include diverse outcomes and engaging, user-centered solutions capable of achieving meaningful improvements at scale.

## Caregivers’ Priorities in Digital Mental Health

Caregivers play an important role in the way teens adopt DMH tools. They prioritize safety, expert validation, and effectiveness [4,5]. Caregivers also value DMH tools that foster positive communication and strengthen the caregiver-teen relationship. Yet, caregivers are challenged with balancing involvement in their teen’s privacy and independence as some teens welcome caregiver involvement while others prefer confidentiality [5,19]. Some research has shown that caregiver involvement in their teen’s DMH treatment actually boosts effectiveness [3]; therefore, it is important to ensure caregiver alignment and participation in the DMH interventions. Concerns about program effectiveness, privacy, and potential technology overuse further complicate caregivers’ support for these tools [20]. Many research-based DMH tools have demonstrated effectiveness in randomized controlled trials [3]. These tools were developed under the oversight of an ethics board and were designed to meet privacy standards such as HIPAA. In contrast, most commercially available DMH interventions for teens have not been rigorously evaluated and are not bound by the same privacy regulations as those in research trials [21]. As such, payors should offer and reimburse for DMH tools that provide evidence-based assurances for caregivers, meet industry privacy standards, and include features that facilitate meaningful caregiver-teen interaction [22,23].

## Teens’ Priorities in Digital Mental Health

Teens engage with DMH tools that benefit them in the moment and are tailored to their unique needs and preferences, making it essential to align their priorities with those of payors and caregivers [24]. Differences in age, cultural background, gender identity, and mental health history can significantly influence how teens understand mental health, what types of support they consider acceptable or trustworthy, and how they engage with digital interventions [25,26]. Acknowledging this diversity helps ensure that DMH tools reflect teens’ varying needs, rather than assuming a one-size-fits-all approach.

DMH tools that feel relatable and foster a sense of connectedness are especially important [3,9,10]. This includes connections with peers who share lived experiences and with supportive professionals [20]. For example, teens using a DMH platform such as Kooth reported reduced psychological distress and loneliness [27]. These teens reported they learned from their peers’ suggestions and experiences and found a sense of purpose by supporting others. Such teen interactions create benefits such as emotional support and peer-driven learning that traditional clinical outcome measures fail to capture. In addition to connectedness, teens also seek DMH tools that provide immediate emotional relief and practical strategies that help them cope [20]. Privacy and anonymity are also important for teen engagement with DMH tools; however, fears of being judged or sharing information can discourage their use [20]. DMH platforms that are confidential and anonymous help to foster the sense of safety that teens need to open up about sensitive topics. Moreover, many teens value the control that DMH apps offer^28^ [29]^30^. Being able to take active steps toward addressing their mental health challenges on their own fosters a sense of agency and empowerment [31]. This sense of control, combined with personalized and immediate support, reflects the outcomes that teens value the most—outcomes that go beyond traditional metrics like symptom reduction and instead emphasize personal growth, emotional connection, and resilience. For payors, recognizing these broader, user-centered outcomes is essential to supporting effective DMH tools. A narrow focus on clinical metrics, such as reductions in depression or anxiety, overlooks the aspects of DMH tools that drive engagement and create meaningful impact for teens. By expanding their perspective to include outcomes that prioritize personalization, privacy, and empowerment, payors can better align their goals with teens’ actual needs, ensuring greater adoption and sustained success of DMH interventions.

## Bridging the Gap: Strategies for Alignment

**Figure 1.**
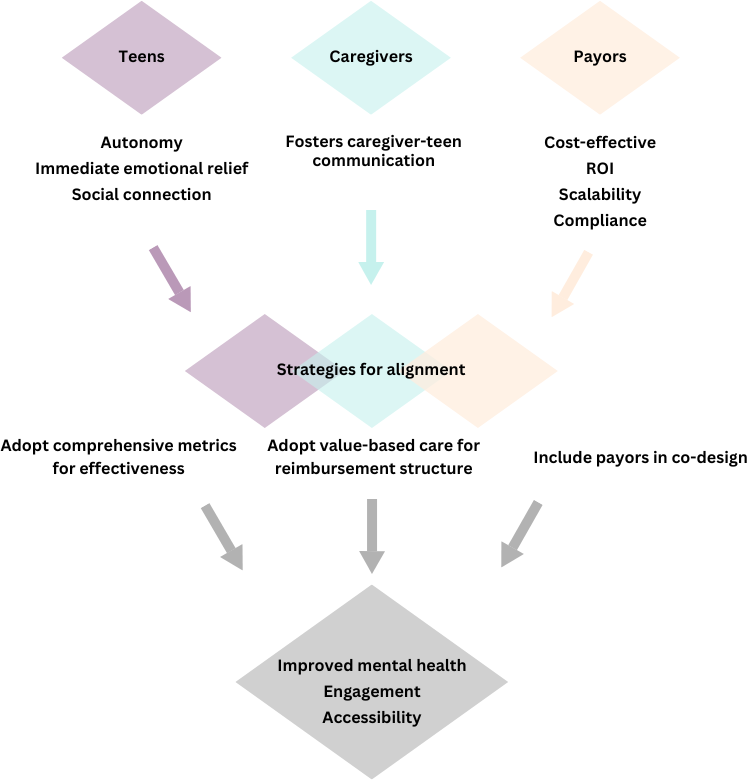
Stakeholder priorities in Digital Mental Health platforms and strategies for alignment. ROI: return on investment.

We suggest several novel but realistic ideas for aligning teen and caregiver priorities with payor priorities (Figure 1). First, we encourage researchers to involve payors in the co-design process of DMH tools. Teen and caregiver perspectives are used to inform product design and they are often included in usability testing, but payor perspectives and feedback are frequently overlooked [32]. Involving payors in product design and gaining their perspective may be an invaluable opportunity to tangibly incorporate their priorities in DMH tools. This information can also serve as a way for researchers and product designers to build relationships with payor stakeholders. If payors were involved in the design of DMH tools, they might better understand how meeting policyholder (ie, teen and caregiver) needs can ultimately meet their own needs, including reducing long-term costs, enhancing satisfaction, and improving member retention.

Another strategy to align competing priorities of teens, caregivers, and payors is to adopt more comprehensive metrics to evaluate the effectiveness and viability of DMH tools. These measures should include traditional symptom assessments (eg, PHQ-9, GAD-7), as well as short, empirically validated user-centric measures such as daily functioning and quality of life [33]. The integration of nonclinical outcomes into reimbursement models should be approached thoughtfully, with careful attention to regulatory guidance and ethical standards, particularly ensuring that such measures are scientifically validated, transparently reported, and equitably applied across populations. Implementing a wider range of metrics requires standardized protocols and pathways for acceptance within reimbursement frameworks, including greater use of existing, validated measures and demonstrating their relevance to outcomes payors currently recognize. Using a multifaceted approach will satisfy the current needs of the payors while beginning to develop an evidence base for outcomes that matter most to teens and caregivers. Over time, this approach could reshape how payors evaluate mental health interventions, shifting focus toward holistic outcomes that align with the real-life priorities of users.

Finally, in our existing health care ecosystem, payors ultimately need evidence that DMH tools are cost-effective and demonstrate a reasonable ROI. One way to bring stakeholders into alignment in this area is to adopt a reimbursement structure for DMH tools that is akin to value-based care. Value-based care is a model that rewards providers for improving outcomes rather than providing services, by, for example, putting a premium on preventive medicine [34]. Value-based care has already been adopted by many payors in traditional healthcare settings. We suggest adopting this model for DMH tools by, for example, encouraging payors to consider tools that impact outcomes tied to early intervention rather than symptom reduction. These might include outcomes that are important for teens and caregivers, such as missed school days, academic performance, and social functioning, as well as outcomes traditionally prioritized by payors, such as emergency department visits and costly prescriptions.

## Conclusion

In conclusion, this paper aimed to outline the priorities of three key stakeholders in the development and delivery of DMH tools for teens: payors, caregivers, and teens. Each of these stakeholder groups has unique priorities that often contradict each other, slowing the development of safe, engaging, accessible, and effective DMH tools for teens. Ultimately, we argued that the needs of caregivers and teens should be met, and that payors should adapt their priorities to align more with the needs of their policyholders. We highlighted feasible ways in which payor needs can more closely align with teens and caregivers, such as including payors in the co-design process, considering broader metrics in determining effectiveness, and adopting a value-based care lens for evaluating the viability of DMH tools for teens. A successful future for DMH tools depends on a collaborative, adaptive approach that prioritizes the unique needs of teens and caregivers while aligning with payor objectives. This synergy can foster the development of solutions that are effective and widely adopted, delivering benefits that resonate with teens and support their long-term well-being.

## Abbreviations

DMH: digital mental health
GAD: General Anxiety Disorder
HIPAA: Health Insurance Portability and Accountability Act
PHQ: Patient Health Questionnaire
ROI: return on investment
